# Anti-tubercular Activity of Pyrazinamide is Independent of *trans-*Translation and RpsA

**DOI:** 10.1038/s41598-017-06415-5

**Published:** 2017-07-21

**Authors:** Nicholas A. Dillon, Nicholas D. Peterson, Heather A. Feaga, Kenneth C. Keiler, Anthony D. Baughn

**Affiliations:** 1 Department of Microbiology and Immunology, University of Minnesota Medical School, Minneapolis, MN 55455 USA; 2 Department of Biochemistry and Molecular Biology, The Pennsylvania State University, University Park, PA 16802 USA

## Abstract

Pyrazinamide (PZA) is a first line anti-tubercular drug for which the mechanism of action remains unresolved. Recently, it was proposed that the active form of PZA, pyrazinoic acid (POA), disrupts the ribosome rescue process of *trans*-translation in *Mycobacterium tuberculosis*. This model suggested that POA binds within the carboxy-terminal domain of ribosomal protein S1 (RpsA) and inhibits *trans*-translation leading to accumulation of stalled ribosomes. Here, we demonstrate that *M. tuberculosis* RpsA interacts with single stranded RNA, but not with POA. Further, we show that an *rpsA* polymorphism previously identified in a PZA resistant strain does not confer PZA resistance when reconstructed in a laboratory strain. Finally, by utilizing an *in vitro trans*-translation assay with purified *M. tuberculosis* ribosomes we find that an interfering oligonucleotide can inhibit *trans*-translation, yet POA does not inhibit *trans-*translation. Based on these findings, we conclude that the action of PZA is entirely independent of RpsA and *trans*-translation in *M. tuberculosis*.

## Introduction

Pyrazinamide (PZA) is a first line anti-tubercular drug that has enabled a reduction in tuberculosis (TB) treatment duration from 9 to 6 months and has played a critical role in lowering relapse rates^1, 2^. Efficacy of PZA is dependent on hydrolysis to pyrazinoic acid (POA) by the *Mycobacterium tuberculosis* encoded pyrazinamidase/nicotinamidase PncA^3^. As PncA is non-essential for growth and pathogenesis of *M. tuberculosis*, the majority of PZA resistant clinical isolates harbor loss-of-function mutations in *pncA* ^4^. While PZA shows sterilizing activity against *M. tuberculosis* in humans and in animal models of TB infection^5–7^, it shows no notable effect on growth of *M. tuberculosis* in standard laboratory culture medium^8^. Since stresses such as acidic pH^9^, nutrient limitation^10^ and anaerobiosis^11^ potentiate the anti-tubercular action of PZA, it has been suggested that this drug selectively targets slow growing and non-growing populations of *M. tuberculosis* ^12^. However, it is important to note that despite this condition-dependence for drug action, PZA and POA do show inhibitory activity against actively replicating *M. tuberculosis* ^10, 13^, indicating that the function(s) disrupted by POA is critical for fitness of the bacilli regardless of their growth status. Understanding the mechanistic basis for action of this important drug will guide discovery efforts for next generation compounds that show improved activity and can circumvent emerging resistance to existing drugs.

Several models have been proposed to explain the anti-tubercular action of PZA. Based on the observation that the action of POA, a weak acid, is enhanced by incubation of mycobacteria under acidic conditions, it was proposed that this drug might act as a proton ionophore causing collapse the cellular membrane potential and intrabacterial acidification^14^. However, since acidic pH is not strictly required for the action of PZA and POA, and treatment with these drugs does not result in rapid disruption of membrane potential nor intrabacterial acidification, ionophoric activity of POA seems insufficient to explain its anti-tubercular action^10, 15^. Another study involving the PZA analog 5-Cl-PZA led to the proposal that POA directly inhibits fatty acid synthase I (FAS-I)^16^. However, while fatty acid synthesis is inhibited following treatment of mycobacteria with PZA and POA, the POA IC_50_ for FAS-I is orders of magnitude greater than that of 5-Cl-PZA^17^. Since PZA and 5-Cl-PZA show similar inhibitory concentrations for susceptible strains of *M. tuberculosis* ^18^, it is reasonable to predict that inhibition of fatty acid synthesis is a downstream consequence of PZA action. Recent molecular studies have established a connection between PZA action and coenzyme A (CoA) metabolism^19–24^. These studies have shown that PZA action can be antagonized by exogenously supplied intermediates of the CoA biosynthetic pathway and that treatment of bacilli with PZA and POA leads to a reduction in CoA abundance^19, 21–23^. Mutations in the carboxy-terminal domain of *panD*, encoding L-aspartate decarboxylase of the CoA biosynthetic pathway, are associated with PZA resistance in broth culture and in the murine model of infection^20, 21, 24^ leading to the suggestion that PanD might be a target of POA. However, a *M. tuberculosis* strain deleted for *panD* still shows conditional susceptibility to PZA^19^, demonstrating that PanD cannot be the exclusive target of this drug. Since CoA is a critical cofactor in fatty acid synthesis, it is likely that disruption of CoA homeostasis explains the ability of PZA to interfere with this pathway. Additional studies are required to delineate the connection between CoA metabolism and PZA action.

In addition to the models described above, it has also been suggested that POA might act by inhibiting *trans*-translation^25^, a process used by bacteria to liberate ribosomes that have stalled on mRNA transcripts lacking an in-frame stop codon^26^. This pathway requires small protein B (SmpB) for recruitment of the dual function transfer-messenger RNA (tmRNA) that promotes release of the stalled ribosome from its non-stop mRNA with subsequent tagging and release of the incomplete nascent peptide^26^. Evidence for POA-mediated inhibition of *trans*-translation in *M. tuberculosis* included *1)* an apparent interaction between POA and ribosomal protein S1 (RpsA) as demonstrated through the use of isothermal titration calorimetry (ITC), *2)* the PZA resistant clinical isolate DHMH444 (PZA MIC of 300 µg/ml) was found to have a polymorphism in *rpsA* (*rpsA*ΔA438; deletion of alanine codon 438), *3)* five-fold over-expression of *rpsA* in *M. tuberculosis* strain H37Ra allegedly conferred resistance to PZA (MIC of 500 µg/ml), and *4)* POA was said to inhibit *trans*-translation in a cell-free assay^25^. As *trans*-translation is important for growth and stress tolerance of many bacteria^27–29^, this model seemed consistent with the ability of PZA to target growing and non-growing populations of *M. tuberculosis*.

Despite its plausibility, this model is inconsistent with previous reports of the role of RpsA in *trans*-translation and the PZA and POA susceptibility phenotypes of *M. tuberculosis* strain DHMH444. While RpsA has been shown to interact with tmRNA, RpsA is not actually required for the *trans*-translation pathway in bacterial systems in which this has been evaluated, such as *Escherichia coli* ^30^ and *Thermus thermophilus* ^31, 32^. Further, a strain of *M. tuberculosis* altered for *trans*-translation showed enhanced susceptibility to antibiotics that interfere with translation, yet, displayed no difference in PZA susceptibility relative to the parental control^33^. In addition, it has been shown that *M. tuberculosis* strain DHMH444 bearing the *rpsA*∆A438 polymorphism is susceptible to PZA in a murine model of infection^34^. While this clinical isolate shows low level resistance to PZA, the role of the *rpsA*∆A438 polymorphism has not been evaluated through the use of matched isogenic strains. This is an important consideration since clinical isolates of *M. tuberculosis* can show over 1,000 genetic polymorphisms^35^. Importantly, strain DHMH444 is known to be fully susceptible to POA in broth culture, and the moderate *in vitro* PZA resistance of this strain has previously been attributed to its reduced PncA activity^36^. Collectively, these observations undermine the likelihood of a role for RpsA and *trans*-translation in PZA action. To resolve these inconsistencies, we further examined the role of RpsA in PZA susceptibility of *M. tuberculosis* and biochemically evaluated the interaction of POA with RpsA and the *trans*-translation complex. Herein we present evidence that *1)* the *rpsA*∆A438 mutation is not associated with PZA resistance, *2)* overexpression of *rpsA* does not confer PZA resistance, *3)* RpsA does not interact with POA, and *4)* POA does not inhibit *trans*-translation. Based on our findings and previously published observations, we conclude that PZA action is entirely independent of RpsA and *trans*-translation in *M. tuberculosis*.

## Results and Discussion

**Figure 1:**
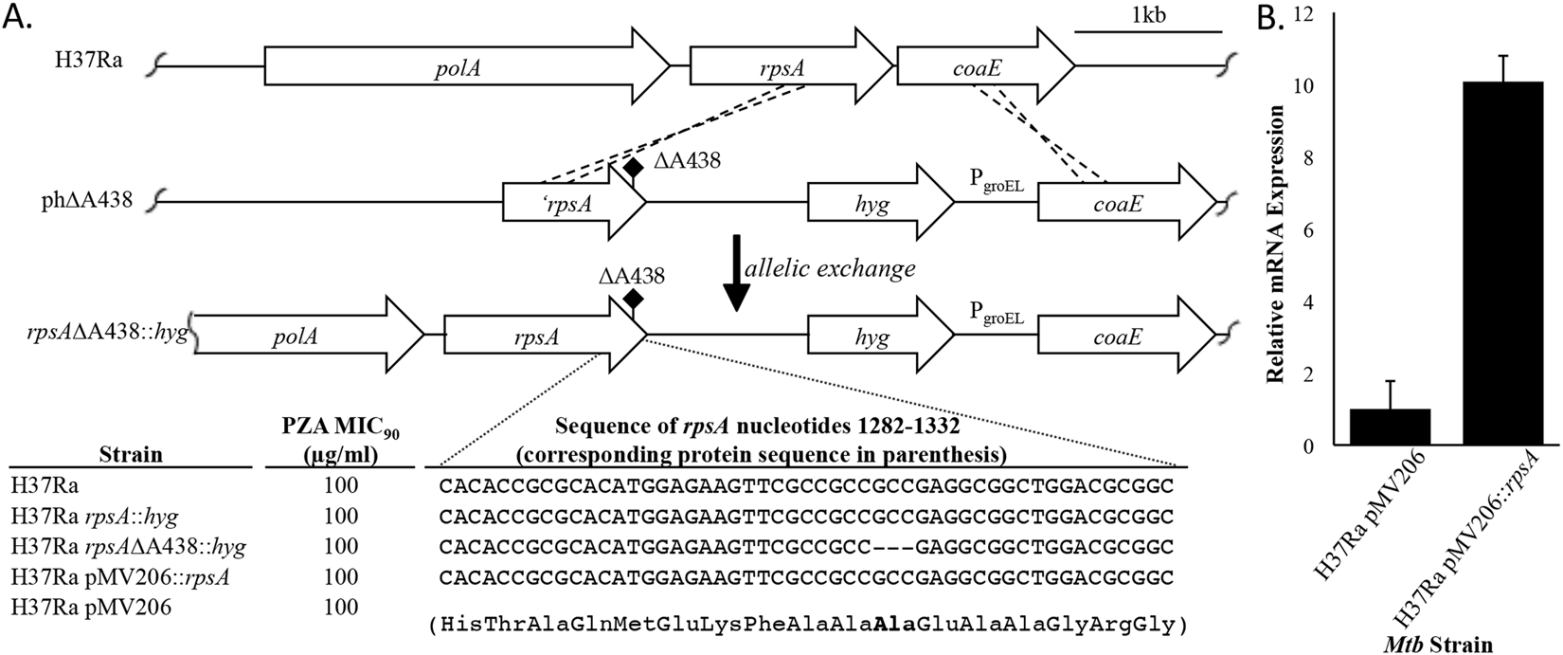
The *rpsA∆*A438 polymorphism and *rpsA* over-expression do not confer PZA resistance in *M. tuberculosis*. (**A**) Schematic representation of specialized linkage transduction used to introduce the *rpsA*∆A438 mutation and corresponding nucleotide sequences of the *rpsA* locus of respective strains. Wild type and *rpsA*∆A438 mutant strains were tested for the minimum concentration of PZA that was required to inhibit at least 90% of growth relative to the no drug control (MIC_90_) over 2 weeks of incubation in supplemented 7H9 medium (pH 5.8). (**B**) Over-expression of *rpsA* in *M. tuberculosis*. qRT-PCR was performed on RNA extracted from *M. tuberculosis* strains H37Ra and H37Ra pMV206::*rpsA*. Relative mRNA expression was calculated using *sigA* transcripts for normalization. All MIC_90_ determinations (**A**) and qRT-PCR assays (**B**) were conducted with at least three independent replicates. Three independent isolates bearing the *rpsA*∆A438 polymorphism, confirmed by full genome sequencing, were assessed. Error bars represent standard deviation from the mean of three independent biological replicates.

Using isogenic *M. tuberculosis* laboratory strains we assessed the impact of the *rpsA*∆A438 polymorphism on PZA susceptibility. Utilizing specialized linkage transduction^37^ we reconstructed the *rpsA*∆A438 polymorphism in the PZA susceptible *M. tuberculosis* laboratory strain H37Ra. Three independent *rpsA*∆A438 and two matched wild type strains were verified by amplifying and sequencing the *rpsA* locus, followed by full genome resequencing. The PZA minimum inhibitory concentration (MIC) for these strains was found to be indistinguishable from that of the parental strain (Fig. 1A). Thus, we conclude that the *rpsA*∆A438 polymorphism is not linked with PZA susceptibility. This observation is not surprising given that the borderline PZA resistant *M. tuberculosis* strain DHMH444 maintains full susceptibility to POA in broth culture^36^ and PZA susceptibility in a murine model of tuberculosis infection^34^. It has been suggested that the low level resistance of this clinical isolate is likely due to its reduced PncA activity^36^. Given the large number of polymorphisms between *M. tuberculosis* clinical isolates and common laboratory strains^35^, it is likely that strain DHMH444 has additional unidentified mutations that are responsible for its low level PZA resistance phenotype.

To further evaluate the connection between RpsA and PZA resistance, *rpsA* was over-expressed in the laboratory strain *M. tuberculosis* H37Ra using an analogous mycobacterial expression vector as previously described^25^. While the previous report did not show data for over-expression of *rpsA* ^25^, quantitative real time PCR (qRT-PCR) from the *M. tuberculosis* strain used herein confirmed 10-fold over-expression of *rpsA* (Fig. 1B). In contrast to the previous report^25^, over-expression of *rpsA* did not confer PZA resistance (Fig. 1A). The basis for this discrepancy in PZA susceptibility with *rpsA* over-expression is unclear, yet, we conclude that PZA susceptibility of *M. tuberculosis* is not affected by abundant over-expression of *rpsA*.

To evaluate whether PZA treatment influences *rpsA* expression levels in *M. tuberculosis*, we reanalyzed transcriptional array data from Boshoff *et al*.^38^. In this previous study, *M. tuberculosis* cultures were treated with a multitude of different anti-tubercular agents and transcriptional microarrays were performed to gain insight on drug action^38^. We re-evaluated data sets from treatment of *M. tuberculosis* with PZA at 1x and 10x the MIC over a time course of 16 hours and found *rpsA* expression levels were relatively unchanged relative to the no-drug control (Supplementary Fig. S1). Thus, PZA treatment does not appear to impact expression of this purported target.

**Figure 2:**
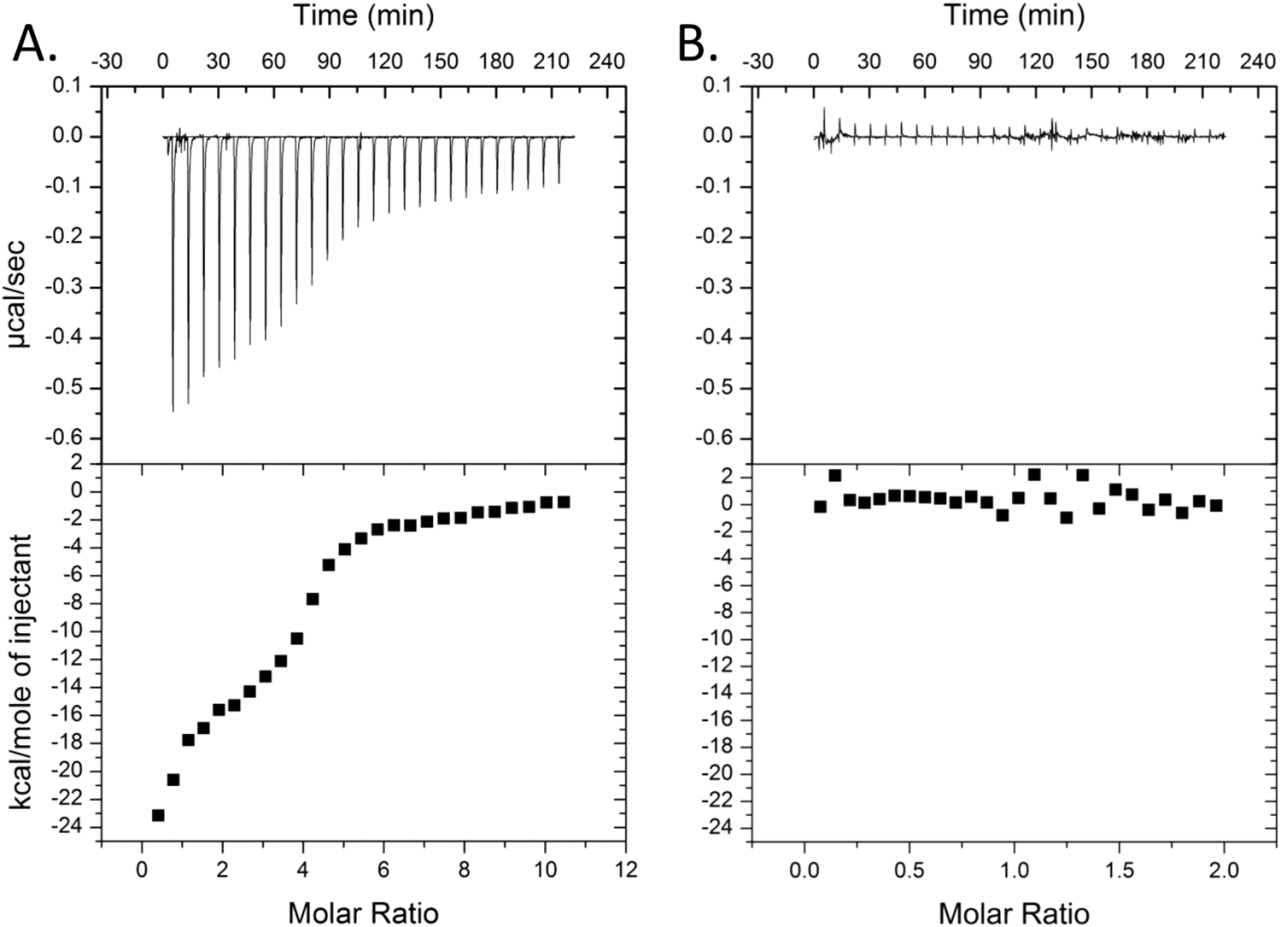
*M. tuberculosis* RpsA interacts with polyC RNA, but not with POA. Isothermal titration calorimetry was used to assess the binding between RpsA and either polyC RNA (**A**) or POA (**B**). In each plot, the top panel represents the heat produced per injection as µcal/sec while the bottom panel shows the change in enthalpy (kcal/mole) as a function of the molar ratio of the two ligands. Titrations were performed at 25 °C using 10 mM phosphate buffer (pH 7.4). POA and polyC RNA solutions were adjusted to pH 7.4.

Next, we utilized isothermal titration calorimetry (ITC) to re-examine the purported interaction between *M. tuberculosis* RpsA and POA. During protein synthesis, RpsA aids in translation initiation via direct binding of single stranded mRNA^39^. *E. coli* RpsA has been shown to have two RNA binding sites with different affinities for polyC RNA^40–42^. Utilizing purified recombinant *M. tuberculosis* RpsA prepared as described in^25^ and polyC RNA as a ligand, we observed a bimodal association curve consistent with the presence of two high-affinity RNA binding sites on RpsA (Fig. 2A). Fitting the data to a multi-binding site model we determined that the higher affinity site of *M. tuberculosis* RpsA bound polyC RNA with a K_*a*_ of 8.91 × 10^7^ ± 5.42 × 10^7^ M^−1^ while the lower affinity site bound polyC RNA with a K_*a*_ of 8.48 × 10^6^ ± 1.85 × 10^6^ M^−1^ (Fig. 2A). These data demonstrate that the purified RpsA is properly folded and has the expected biochemical properties. Despite this robust interaction with polyC RNA and in contrast to that which was described previously^25^, no signal was observed when POA was titrated with RpsA (Fig. 2B). These data demonstrate that *M. tuberculosis* RpsA and POA do not show detectable interaction.

**Figure 3:**
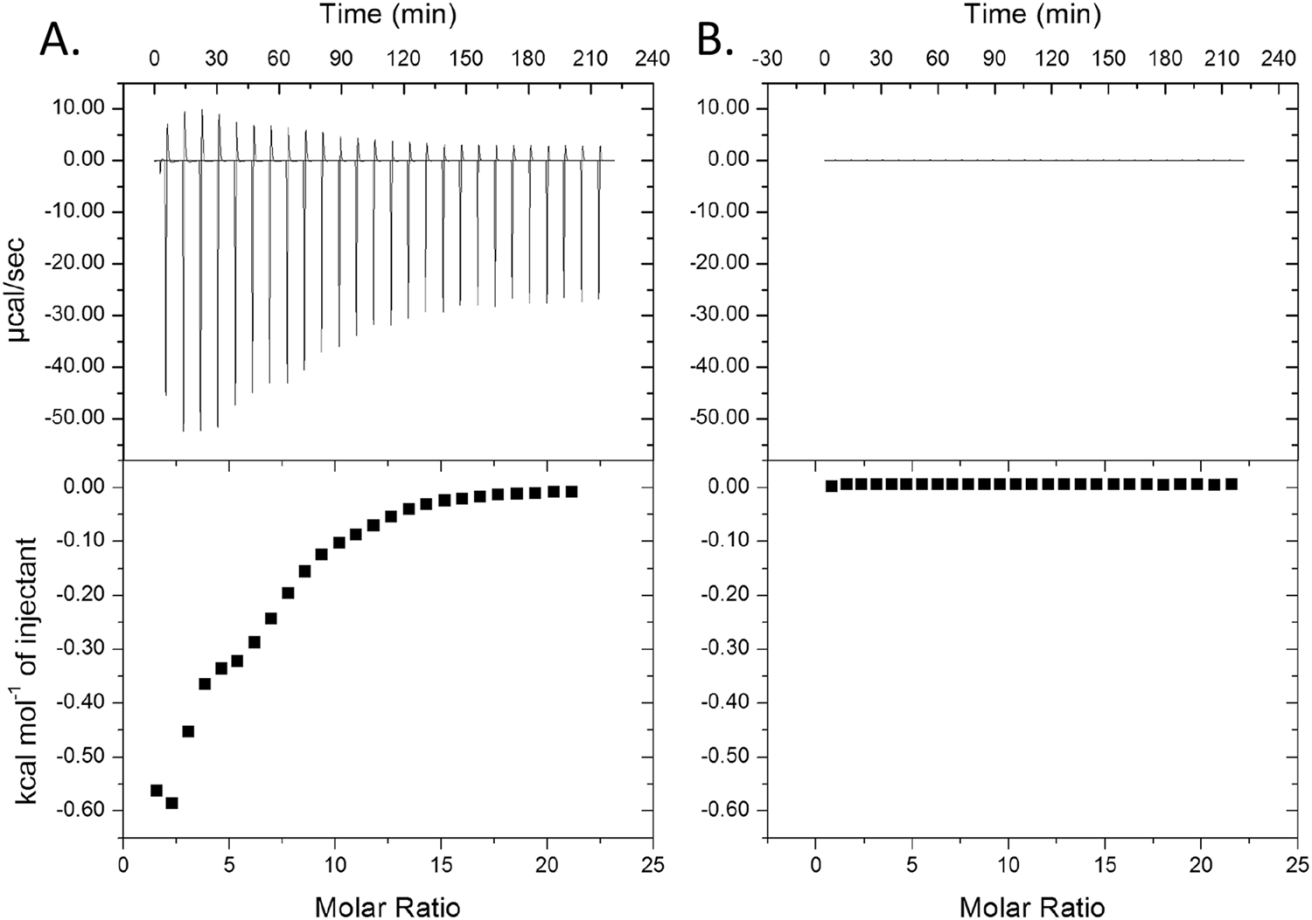
Unbuffered POA gives a robust ITC signal when titrated into neutral buffer. Saturated POA ((**A**) unadjusted pH 2.3; (**B**) pH adjusted to 7.4) was injected into 10 mM phosphate buffer (pH 7.4).

ITC is a sensitive approach for measurement of thermodynamic reactions in solution. Injection of a weak acid, such as POA (pK_a_ of 2.9), into a modestly buffered solution can generate a robust signal due to pH dependent proton dissociation. In the previous study, a saturated solution of POA (~70 mM) was used for evaluating interaction with recombinant *M. tuberculosis* RpsA, and was erroneously compared to titrations involving 100 µM POA with recombinant RpsA from either *M. tuberculosis* strain DHMH444 or *M. smegmatis* ^25^. As anticipated, we find that when a saturated POA solution is titrated into 10 mM phosphate buffer, a robust signal due to proton dissociation is observed and is entirely independent of the presence of RpsA (Fig. 3A). This signal is fully abrogated when the pH of the saturated POA solution is adjusted to that of the diluent buffer (Fig. 3B), confirming that this robust signal is the result of acid dissociation. As the authors of the previous report describe using saturating levels of POA when signal was observed and ~700 fold less POA when signal was not observed, we speculate that the reported signal for *M. tuberculosis* RpsA and POA was not due to interaction between these ligands, but was instead due to heat of proton dissociation of the weak acid POA.

These ligand interaction studies call into question the initial basis for identification of RpsA as an interaction partner of POA. In the previous study^25^, the authors utilized a column in which 5-hydroxy-POA was covalently coupled to a Sepharose resin to purify proteins from a cellular lysate of *M. tuberculosis* strain H37Ra. Rather than utilizing free POA to elute proteins that bound specifically to the POA moiety, bound proteins were stripped from the column with 25% ethylene glycol. Through this approach, several proteins were obtained, including Rv2783c, RpsA, Rv2731, Rv3169, and others that were not specifically identified. As RpsA is one of the most abundant proteins in the *M. tuberculosis* proteome^43^ and this purification approach resulted in isolation of numerous proteins, we speculate that identification of RpsA was merely due to its high abundance in the cellular extracts that were used.

**Figure 4:**
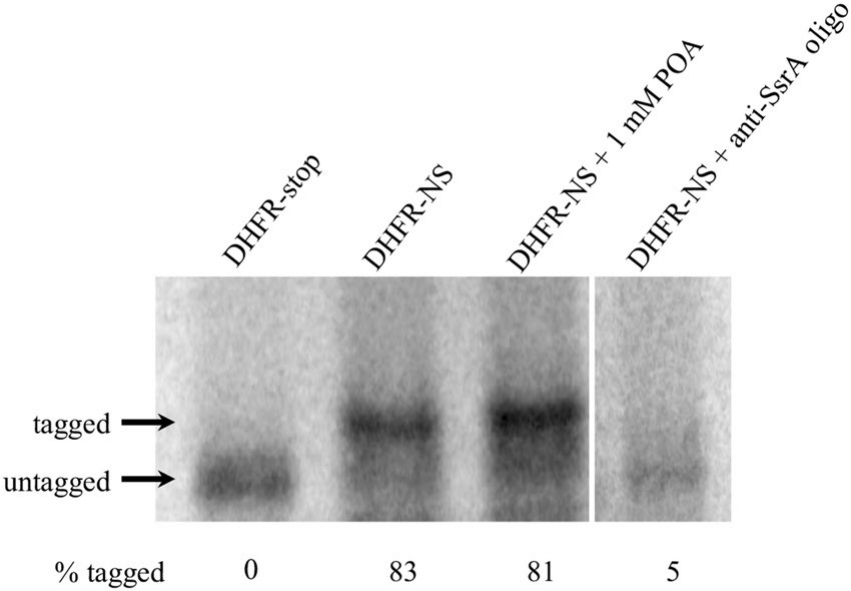
POA does not inhibit *trans*-translation *in vitro*. *trans*-Translation reactions with 50 nM *M. tuberculosis* ribosomes, 150 nM *M. tuberculosis* tmRNA, 150 nM *M. tuberculosis* SmpB, and 640 nM template DNA. Where indicated pyrazinoic acid (Sigma) was added to 1 mM or anti-SsrA oligonucleotide was added to 2 µM. Reactions were incubated at 37 °C 3 h and analyzed by SDS-PAGE followed by phosporimaging. This image was cropped to improve clarity. A full-length image is provided as Supplementary Figure S2.

To specifically address whether POA can inhibit the tagging activity mediated by the *trans*-translation pathway, we utilized an *in vitro trans*-translation assay^44^. A gene encoding dihydrofolate reductase (DHFR) lacking a stop codon (DHFR-NS) was used as a template in a transcription/translation assay containing *M. tuberculosis* ribosomes, tmRNA and SmpB. Expression of DHFR-NS with *M. tuberculosis* components resulted in tagging of the DHFR-NS demonstrating that *trans*-translation functions *in vitro* with *M. tuberculosis* components (Fig. 4). Incubation with 1 mM POA resulted in no substantial reduction in the tagging activity of *trans*-translation (1.6 ± 4%), while incubation with an anti-sense SsrA oligonucleotide exhibited >90% reduction in tagging activity (Fig. 4). These observations lead us to conclude that POA does not inhibit *trans*-translation in *M. tuberculosis*.

These results conflict with those described in Shi *et al*.^25^. There are several important differences between the assay conditions that likely account for the discrepant observations. First, the previous study used a DHFR gene that was followed by 26 additional codons (8 rare AGG codons and 18 additional codons) and a stop codon as the template for expression. Although expression of genes containing rare codons can lead to *trans*-translation in *E. coli* cells, the mRNA must first be cut by a nuclease before *trans*-translation can occur^45^. No RNases were included in the reactions described in^25^, so expression of the gene used in those reactions would not induce a high rate of *trans*-translation and instead would lead to production of DHFR containing the additional 26 template encoded amino acids. Second, in Shi *et al.* ^25^ pre-tmRNA was added to reactions mixtures and did not contain the enzymes required for pre-tmRNA processing and maturation^46–49^. Pre-tmRNA cannot be charged with alanine and is inactive for *trans*-translation^49^. Despite using inactive tmRNA and a gene that would not result in a high rate of *trans*-translation, Shi *et al*.^25^ report 100% tagged protein in the absence of POA, and observe complete inhibition of protein synthesis with ≥25 µg/ml POA using their DHFR-8xAGG gene. It is important to note that by the nature of this assay, inhibition of *trans*-translation would still result in production of a band corresponding to untagged DHFR as ^35^S-methionine is incorporated during translation of the nascent peptide regardless of subsequent engagement of *trans*-translation^50^. In other words, translation is a prerequisite for *trans-*translation and failure to observe untagged protein is indicative of inhibition of translation. However, failure to observe signal could also be explained by failure to load sample in the respective lanes. Regardless, the most likely explanation for the observations in the previous study is that the authors did not correctly assign the DHFR bands in their figure and no *trans*-translation occurred in their assays. In contrast, in our study, the assays shown in Fig. 4 use mature tmRNA and a template gene with no stop codon, which has been shown to induce *trans*-translation^44^. The anti-SsrA oligo control in Fig. 4 demonstrates that the change in mobility of the protein product we observed is due to *trans*-translation.

Collectively, our observations demonstrate that RpsA and *trans*-translation do not have a role in the mode of action of PZA and that the mechanistic basis for PZA susceptibility remains to be elucidated. Given the recent observations by multiple independent laboratories that PZA action can be antagonized by intermediates of the CoA biosynthetic pathway and that alterations in CoA metabolism influence PZA susceptibility^19–24^, it is likely that this drug directly impairs a critical player in CoA metabolism. Interestingly, mutations in the gene for the ClpC1 unfoldase have also recently been recognized in *M. tuberculosis* PZA resistant laboratory isolates^24, 51, 52^, yet, the mechanistic basis for the association between ClpC1 and PZA resistance remain unclear. Considering the unparalleled *in vivo* sterilizing activity of PZA and the increasing global burden of multidrug-resistant and extensively drug-resistant tuberculosis, elucidating the requirements for susceptibility of *M. tuberculosis* to key drugs such as PZA is of paramount importance for the optimization of impactful treatment regimens.

## Materials and Methods

### Bacterial strains and growth conditions

*Mycobacterium tuberculosis* strain H37Ra was grown in Middlebrook 7H9 medium (Difco) supplemented with 10% (vol/vol) oleic acid-albumin-dextrose-catalase (OADC) (Difco), 0.2% (vol/vol) glycerol, and 0.05% (vol/vol) tyloxapol. *Escherichia coli* strains DH5α used for the propagation of phasmids and plasmids and BL21(DE3) used for overexpression and purification of protein were grown in Lysogeny Broth (LB). Antibiotics hygromycin (150 μg ml^−1^) and kanamycin (50 μg ml^−1^) were used as necessary.

### RpsA Expression and Purification

For RpsA expression and purification, *M. tuberculosis* encoded *rpsA* was amplified by PCR, digested with *Bam*HI and *Hin*dIII, and ligated into pET-28b+ digested with the same enzymes. The recombinant plasmid was transformed and propagated in BL21(DE3) selected with kanamycin. Growing BL21(DE3) containing pET-28b+*rpsA* was induced with 0.25 mM isopropyl β-D-1-thiogalactopyranoside (IPTG) for 3 hours at 37 °C. Induced cells were harvested via centrifugation and resuspended in 50 mM NaH_2_PO_4_, 300 mM NaCl, 10 mM imidazole pH 8.0. Cells were disrupted by sonication and debris pelleted via centrifugation. Supernatant was bound to Ni^2+^-NTA resin (Qiagen), previously washed and equilibrated with 50 mM NaH_2_PO_4_, 300 mM NaCl, 20 mM imidazole pH 8.0, by mixing at 4 °C. Resin supernatant slurry was packed into a chromatography column and washed with 20 column volumes of 50 mM NaH_2_PO_4_, 300 mM NaCl, 20 mM imidazole pH 8.0. Bound RpsA was eluted with 5 column volumes of 50 mM NaH_2_PO_4_, 300 mM NaCl, 250 mM imidazole pH 8.0. Eluted RpsA was dialyzed with 800 ml 10 mM phosphate buffer twice for 3 hours and once for 16 hours. Overexpression and purification were confirmed via SDS-PAGE gel.

### Isothermal titration calorimetry (ITC) assay

The ITC interaction assays were conducted on the MicroCal VP-ITC at 25.0 °C. POA, RpsA, and the polyC-RNA positive control were dissolved in 10 mM phosphate buffer (pH 7.4). The pH of the POA buffer solution was adjusted to pH 7.4 to account for any pH change due to POA, unless otherwise indicated. The drugs were loaded into the syringe at the indicated concentrations. Each experiment consisted of 26 10 μl injections over a 2 second duration into 1449.7 μl of ligand within the cell. The solution mixtures were stirred at 300 rpm and the interval between injections was set at 500 seconds. Origin 7 software (Origin®) was used to collect and analyze the data.

### qRT-PCR

For quantification of *rpsA* overexpression quantitative reverse transcription-PCR (qRT-PCR) was performed. Briefly, mid exponential phase *M. tuberculosis* was harvested via centrifugation. Cell pellet was resuspended in 100 μl 10 mM Tris-HCl, 1 mM EDTA, 15 mg/ml lysozyme and incubated at 37 °C for 16 hours. RNA was extracted using the E.Z.N.A.^TM^ bacterial RNA kit (Omega Biotek). Remaining DNA was removed by treatment with TURBO DNA-free^TM^ kit (Ambion). Gene specific primers for qRT-PCR were designed with Primer3 software. qRT-PCR was performed with the QuantiFast^®^ SYBR^®^ Green RT-PCR kit (Qiagen). qRT-PCR reactions were prepared with 2X QuantiFast SYBR Green RT-PCR master mix, 10 μM primers, 0.1 μl QuantiFast RT Mix, 1 ng RNA and were run on a LightCycler^®^480 with following cycle conditions: 50 °C for 10 min, 95 °C for 5 min, 35 cycles of 95 °C for 10 s, 60 °C for 10 s, and 72 °C for 20 s with fluorescence quantification each cycle. Melting curve cycle of 95 °C for 15 s, 60 °C for 15 s, and 95 °C with 2% ramp rate to determine product specificity. qRT-PCR reactions lacking reverse transcriptase were performed to test for contaminating DNA.

### Cloning of allelic exchange and overexpression strains

To generate the *rpsA* ∆A438 polymorphism in *M. tuberculosis rpsA* was first cloned into p0004s using PCR amplification followed by a 4 piece ligation. The 5′ piece was amplified with the primers *rpsA*_5′_F and *rpsA*_5′_R and digested with *Pac*I and *Nhe*I. The 3′ piece was then amplified via PCR with the primers *rpsA*_3′_F and *rpsA*_3′_R and digested with *Nco*I and *Nde*I. The primers designed for the *rpsA*∆A438 quick change, *rpsA*∆A438_F and *rpsA*∆A438_R, were then used to introduce the alanine deletion in *rpsA* via PCR amplification. The resulting cosmid was then inserted into phAE159 and was introduced into *M. tuberculosis* via specialized transduction.

For RpsA overexpression, *M. tuberculosis* encoded *rpsA* was amplified by PCR with primers indicated in Table S1, digested with *Nhe*I and *Xba*I, and ligated into mycobacterial replicative expression vector pMV261 digested with *Xba*I. The recombinant plasmid was transformed and propagated in DH5α selected with kanamycin. For RpsA overexpression H37Ra was electroporated with the *rpsA* overexpression plasmid and selected on supplemented 7H10 containing kanamycin.

### Pyrazinamide susceptibility testing

Antimicrobial susceptibility was determined by measuring optical density of respective cultures at 600 nm (OD_600_). PZA susceptibility testing was performed using supplemented 7H9 medium adjusted to pH 5.8. The minimum inhibitory concentration (MIC_90_) for antimicrobial compounds was defined as the minimum concentration required for inhibition of at least 90% of growth, relative to the no antimicrobial control. Growing *M. tuberculosis* H37Ra was diluted to an OD_600_ of 0.01 in 5 ml of supplemented 7H9 medium in 30 ml square Nalgene bottles. Antimicrobial compounds were added to the final concentrations indicated in the text. Cultures were incubated at 37 °C with shaking on a rotary platform at 100 rpm for 14 days. All results presented are from a minimum of three independent determinations.

### Ribosome purification

Cells were grown in supplemented 7H9 until mid exponential phase (OD_600_ of 0.5) and subjected to French pressure lysis. The lysate was cleared by centrifugation at 30,000 × g for 20 min, and crude ribosomes were harvested from the supernatant by centrifugation at 100,000 × g for 2 h. The pellet was washed 3 X in Buffer II (20 mM Tris-HCl [pH 7.6], 1 M ammonium acetate, 10 mM magnesium acetate, 6 mM ß-ME), and resuspended in Buffer I (10 mM Tris-HCl [pH 7.6], 100 mM ammonium acetate, 10 mM magnesium acetate, 6 mM β-ME). 70S ribosomes were isolated by sucrose density fractionation (10–40% sucrose in 10 mM Tris-HCl [pH 8.0], 30 mM KCl, 10 mM magnesium acetate).

### Isolation of *M. tuberculosis* tmRNA and SmpB

*M. tuberculosis ssrA* was amplified by PCR using primers MtbSsrAF and MtbSsrAR to place it under control of a T7 promoter. The product was gel purified and used as template in a second PCR reaction with primers MtbSsrAF and MtbSsrAR. The product was transcribed *in vitro* and purified as described previously for *E. coli* tmRNA^44^. *M. tuberculosis smpB* was amplified by PCR using primers TB_SmpB_F and TB_SmpB_R, digested with *Hin*dIII and *Nde*I, and ligated into pET28b that had been digested with the same enzymes. SmpB was produced and purified as described previously for *E. coli* SmpB^44^.

### *In vitro trans-*translation assays

Template construction and reaction conditions were as previously described^44^, with the following modifications. DHFR-stop and DHFR-NS templates were made by PCR using T7 universal primer and either Stop_UTR_DHFR_FL or NS_UTR_DHFR_FL. *trans*-translation reactions used the PURExpress ribosome-free kit (New England Biolabs) with 50 nM *M. tuberculosis* ribosomes, 150 nM *M. tuberculosis* tmRNA, 150 nM *M. tuberculosis* SmpB, and 640 nM template DNA. Where applicable, pyrazinoic acid (Sigma) was added to 1 mM or anti-SsrA oligo was added to 2 µM. Reactions were incubated at 37 °C 3 h and analyzed by SDS-PAGE followed by phosporimaging^44^.

### Data avai﻿lability

The datasets generated during and/or analyzed during the current study are available from the corresponding author on reasonable request.

## Electronic supplementary material

Supplemental Information
