# Supplementary material for: Anti-tubercular Activity of Pyrazinamide is Independent of trans-Translation and RpsA
Source: Sci Rep. 2017 Jul 21;7:6135. doi: 10.1038/s41598-017-06415-5 (PMC5522395; doi:10.1038/s41598-017-06415-5)
Supplement: Supplementary file 1 — Supplemental Information [file 41598_2017_6415_MOESM1_ESM.pdf]

Anti-tubercular Activity of Pyrazinamide is Independent of *trans*-Translation and RpsA

Nicholas A. Dillon[1†], Nicholas D. Peterson[1†], Heather A. Feaga[2], Kenneth C. Keiler[2], and Anthony D. Baughn[1*]

[1]*Department of Microbiology and Immunology, University of Minnesota Medical School, Minneapolis, MN 55455.* [2]*Department of Biochemistry and Molecular Biology, The Pennsylvania State University, University Park, PA 16802.*

*Correspondence to: Anthony D. Baughn, abaughn@umn.edu.

†Contributed equally to this work.

Table S1. Oligonucleotide primers used in this study.

Name of oligonuleotide	Sequence 5' to 3'
MtbSsrAF	GAAATTAATACGACTCACTATAGGGGCTGAACGGTTTCGACTT
MtbSsrAR	TGGTGGAGCTGCCGGGAATC
TB_SmpB_F	CGCGGCAGCCATATGGTGTCCAAGTCGTCGCGTG
TB_SmpB_R	CTCGAGTGCGGCCGCAAGCTTCAGGTCATGCCCTTAGCGC
Anti-SsrA	ATGTGAATCGGCGCTTATT
*rpsA*_5'_F	TTAATTAAACATCGGCAAGGAGATCGAG
*rpsA*_5'_R	GCTAGCTCAAGCGCTGCCGGCGAGTTTTTCCC
*rpsA*_3'_F	CCATGGGCCGGCAGCGCTTGATCTTG
*rpsA*_3'_R	CATATGTCGTTCAGGGACGCCAGTG
*rpsA*ΔA438_F	GAGGCGGCTGGACGCGGCGCGGACGATCAGTC
*rpsA*ΔA438_R	GGCGGCGAACTTCTCCATCTG

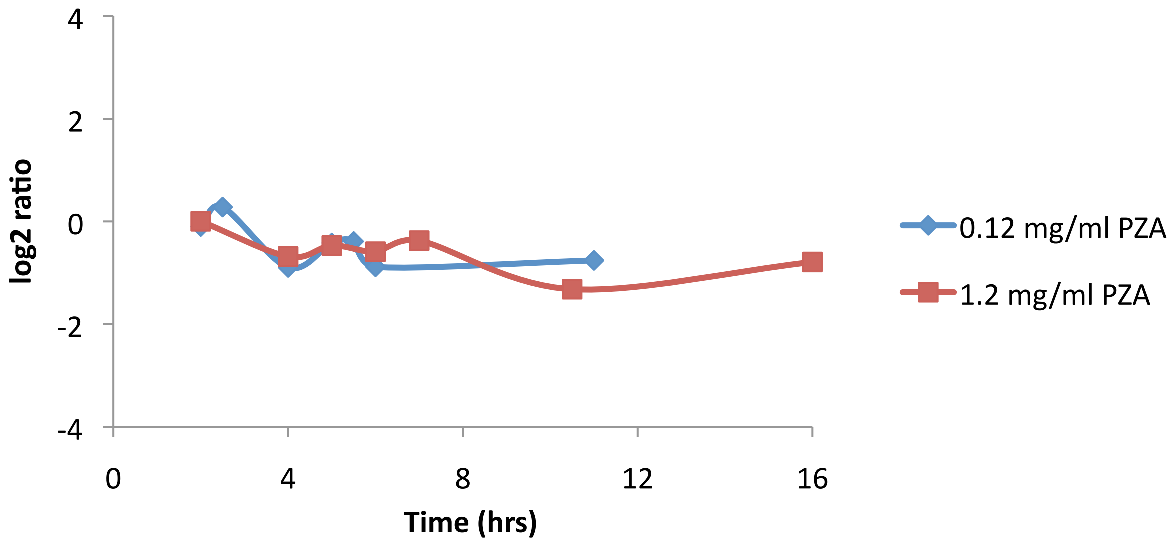

Figure S1. PZA does not impact *rpsA* expression levels in *M. tuberculosis*. Data represent microarray log 2 signal intensities for *rpsA* from *M. tuberculosis* cultures that were treated with 0.12 mg/ml PZA or 1.2 mg/ml PZA relative to the no PZA control from Boshoff *et al.* (2004).

Boshoff, H.I.M. *et al.* The Transcriptional Responses of *Mycobacterium tuberculosis* to Inhibitors of Metabolism: NOVEL INSIGHTS INTO DRUG MECHANISMS OF ACTION. *J Biol Chem* **279**, 40174-40184 (2004).

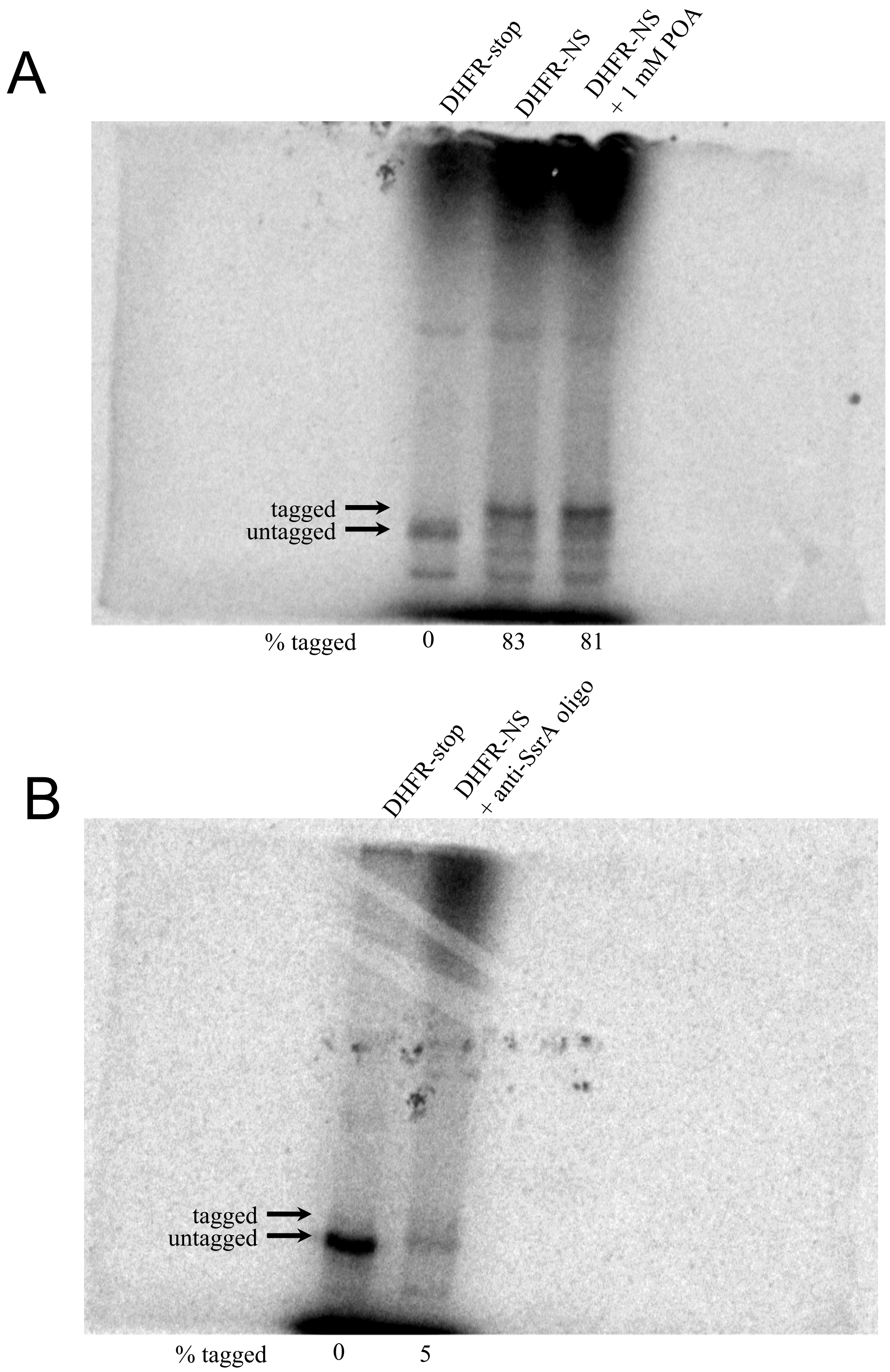

Figure S2. POA does not inhibit *trans*-translation *in vitro*. Original uncropped images from Figure 4. Panel A, *in vitro* translation of non-tagged protein (DHFR-stop), *trans*-translation of SsrA tagged protein from a non-stop template (DHFR-NS), *trans*-translation of DHFR-NS despite addition of 1 mM POA to the reaction mixture (DHFR-NS + 1 mM POA). Panel B, *in vitro* translation of non-tagged protein (DHFR-stop) and inhibition of *trans*-translation with 2 μM anti-SsrA oligonuleotide (DHFR-NS + anti-SsrA oligo). *trans*-Translation reactions were carried out with 50 nM *M. tuberculosis* ribosomes, 150 nM *M. tuberculosis* tmRNA, 150 nM *M. tuberculosis* SmpB, and 640 nM template DNA. Reactions were incubated at 37 °C 3 h and analyzed by SDS-PAGE followed by phosphorimaging.
